# Coronary Steal in a Patient With Apical Hypertrophic Cardiomyopathy: A Rare Case of Symptomatic Coronary Artery Fistula

**DOI:** 10.7759/cureus.11793

**Published:** 2020-11-30

**Authors:** Neil R Patel, Sangeeta Prabhakar Bhat, Shantanu Solanki, Terry Bauch, Yassir Nawaz

**Affiliations:** 1 Cardiology, The Wright Center for Graduate Medical Education, Scranton, USA; 2 Internal Medicine, The Wright Center for Graduate Medical Education, Scranton, USA; 3 Hospital-Based Medicine, Geisinger Commonwealth School of Medicine, Scranton, USA; 4 Cardiology, Geisinger Wyoming Valley Medical Center, Wilkes Barre, USA; 5 Cardiology, Geisinger Community Medical Center, Scranton, USA

**Keywords:** congenital anomalies of coronary arteries, coronary artery fistula

## Abstract

This report describes a rare case of multiple left coronary artery to pulmonary artery/left atrial fistulae causing a coronary steal phenomenon. A 58-year-old male with apical hypertrophic cardiomyopathy was seen in an outpatient office for exertional chest pain and dyspnea and subsequently had a positive exercise nuclear stress test. Coronary angiogram revealed 70-80% mid-left anterior descending artery stenosis with multiple proximal coronary artery to left atrial/pulmonary artery fistulae. Due to symptomatic coronary artery fistulae with coronary steal phenomenon, the patient underwent surgical correction of fistulae with bypass graft to left anterior descending artery. To our knowledge, this is the first case report on co-existing apical hypertrophic cardiomyopathy and coronary artery-left atrial/pulmonary artery fistulae. This article reviews current guidelines for management of coronary artery fistula.

## Introduction

Coronary artery fistulae (CAF) are abnormal communications between the coronary arteries and cardiac chambers or great vessels which are incidentally found in patients undergoing coronary angiography. Incidence of CAF has been reported about 0.2% [1]. There are very few case reports on apical hypertrophic cardiomyopathy (AHCM) coexisting with coronary artery fistula. Most of these reports are on coronary artery to left ventricle fistula [2,3]. We are reporting a rare case of symptomatic coronary artery to left atrium and pulmonary artery fistula coexisting with apical hypertrophic cardiomyopathy.

## Case presentation

A 58-year-old Caucasian male presented to the outpatient cardiology clinic with exertional chest heaviness associated with shortness of breath. His symptoms were exacerbated by increased activity such as mowing the lawn or climbing up a flight of stairs and relieved after a few minutes of rest. He did not complain of palpitations, leg swelling, dizziness, or syncope. Family history was significant for premature coronary artery disease in his father. Home medications included metoprolol succinate, aspirin, and atorvastatin. Review of systems was otherwise unremarkable. On physical examination, his vitals were stable. He had a normal body mass index of 27.2 kg/m2. Physical examination was benign without any cardiac murmurs or signs of heart failure. 

**Figure 1:**
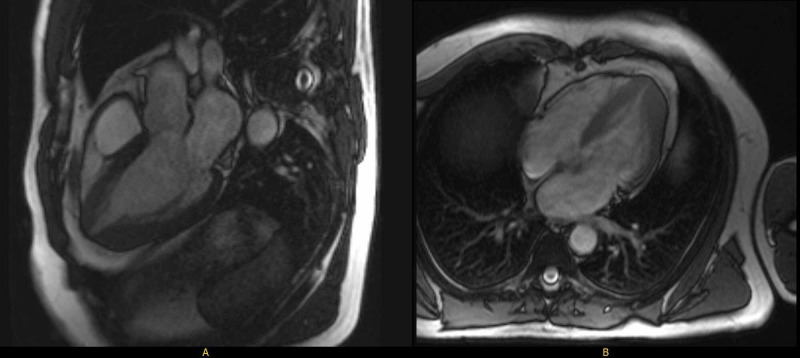
Cardiac MRI showing apical hypertrophy with 17 mm thickness of apex

The patient was diagnosed with AHCM after a transthoracic echocardiogram in 2015 revealed asymmetric apical hypertrophy. Cardiac MRI in 2016 confirmed AHCM with apical thickness of 17 mm and minimal atypical delayed enhancement of the thickest area of the apex (Figure 1). Exercise electrocardiography in 2018 was unremarkable. His chronic hepatitis C was treated three years ago. He had a five-pack-year history of smoking and had quit 40 years ago.

**Figure 2:**
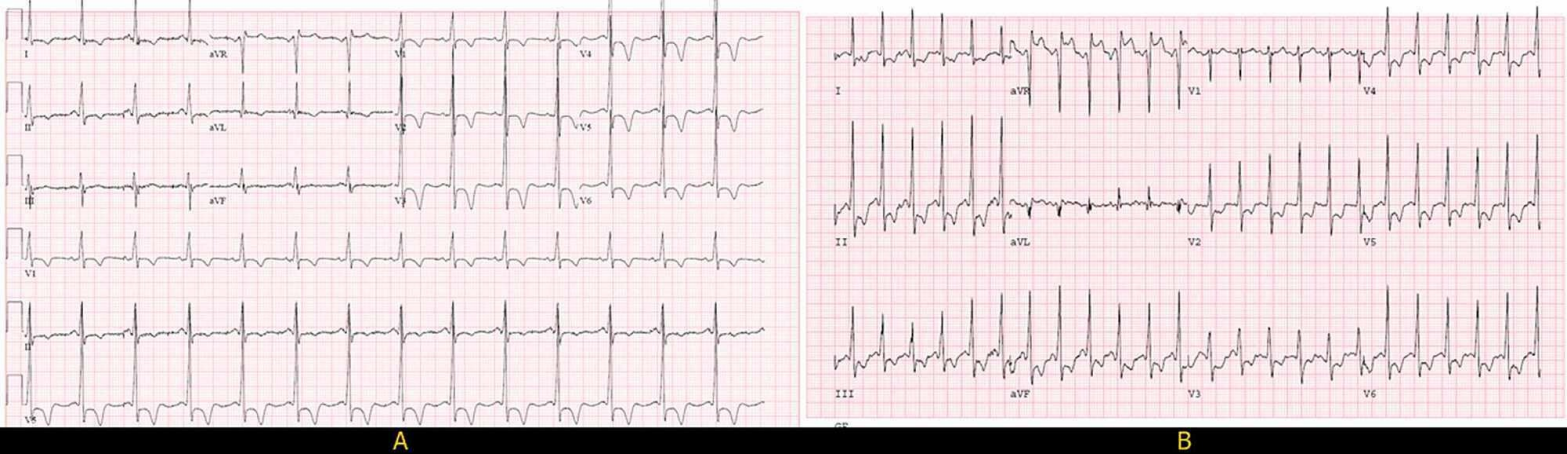
Baseline and Stress Electrocardiogram (EKG) A) Baseline EKG: Normal Sinus rhythm with left ventricular hypertrophy with precordial deep T wave inversion. B) Peak stress EKG (6-minute) showing more pronounced ST depression in precordial and inferior leads.

**Figure 3:**
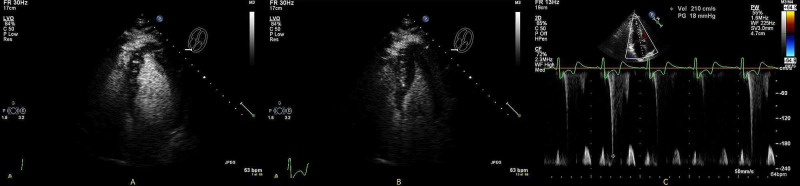
Transthoracic echocardiogram showing apical hypertrophy Apical 4-chamber view in end-diastole (A) and at end-systole (B) showing characteristic “ace of spades” appearance with narrowing of left ventricular apex. (C) Pulsed wave doppler tracing at left ventricular apex demonstrating pressure gradient.

**Figure 4:**
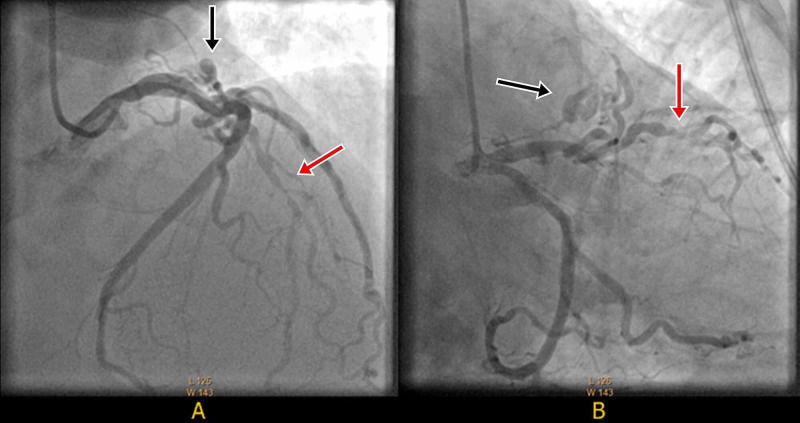
Left coronary angiogram AP Cranial view (A) and RAO Caudal view (B) showing 70-80% mid-distal LAD artery stenosis (red arrow) with large fistula from proximal LAD to left atrium (black arrow). AP= Antero-posterior, RAO=Right anterior oblique, LAD= Left anterior descending.

**Figure 5:**
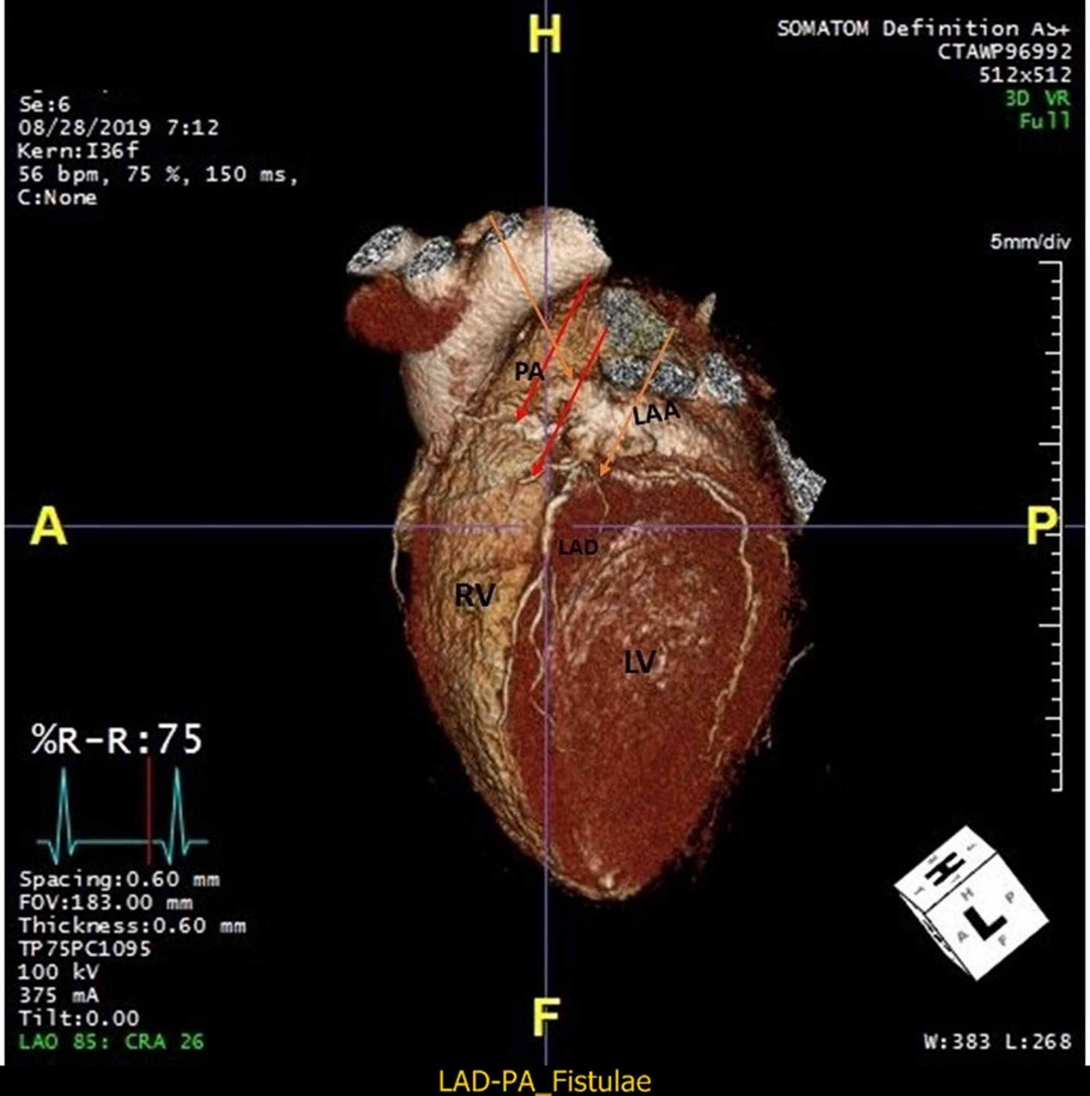
Cardiac CTA image showing CAF 3D image showing multiple proximal LAD fistulae draining into left atrial appendage and pulmonary artery. (Red and orange arrow). CAF= Coronary artery fistulae, LAD= left anterior descending

An electrocardiogram (EKG) done in the clinic was significant for left ventricular hypertrophy with deep T wave inversions in the anterolateral leads unchanged from previous (Figure 2A). Exercise myocardial perfusion imaging revealed a medium-sized completely reversible apical anterior perfusion defect suggestive of stress-induced ischemia. More than 2 mm horizontal ST-T depressions over baseline ST-T changes were noted (Figure 2B). His echocardiogram showed normal left ventricular ejection fraction with asymmetrical hypertrophy of apex, normal size of both atria, mild mitral and tricuspid regurgitation, normal right ventricular size and function, and normal estimated pulmonary artery systolic pressure (Figure 3). Laboratory tests including blood counts and renal function panel prior to cardiac catheterization were unremarkable. Left heart catheterization revealed severe one vessel coronary artery disease with mid to distal left anterior descending (LAD) artery stenosis of 70-80%. A large fistula between LAD and probably left atrium was noted. There was prominent blood flow into the fistula with decreased blood flow in the LAD suggestive of a steal phenomenon (Figure 4, Video 1). Left ventricular end-diastolic pressure (LVEDP) was reported as normal. He subsequently had cardiac CT angiogram (CCTA) which confirmed presence of left coronary artery fistula originating from two locations in the proximal LAD, just proximal and distal to the first diagonal branch. The fistulae drained into the left atrial appendage and possibly into the pulmonary artery through multiple small caliber sinusoidal connections (Figure 5).

**Video 1:** Coronary angiogram RAO Caudal view showing 70-80% mid-distal LAD artery stenosis (red arrow) with large fistula from proximal LAD to left atrium (black arrow) causing coronary steal phenomenon. RAO=Right anterior oblique, LAD= Left anterior descending.

Due to symptomatic coronary artery fistula with coronary steal phenomenon, it was deemed best for the patient to undergo surgical correction of the fistulae with bypass graft to LAD. Cardiothoracic surgery was consulted. During the operative procedure, he was noted to have three fistulae between the proximal LAD draining into the left atrial appendage and pulmonary artery. He underwent ligation of the fistulae with a free right internal mammary artery graft to the mid LAD. The coronary artery bypass surgery was uneventful and the patient was discharged to home. He is currently free of symptoms and continues to be on antiplatelet therapy. 

## Discussion

CAF are rare congenital or acquired coronary artery abnormalities in which blood is shunted into a cardiac chamber, great vessel or other structure, bypassing the myocardial capillary network [4]. CAF are incidentally found in 0.25% of patients undergoing coronary angiography [5]. CAF commonly terminate in the right ventricle, right atrium, and pulmonary artery, being low-pressure chambers [1]. Our patient was found to have a unique CAF draining into the left atrium and pulmonary artery. 

The clinical and hemodynamic consequences of CAF are not completely understood. Due to highly variable clinical presentation, it is almost always incidentally identified. Most CAF are asymptomatic due to their small size but hemodynamically significant fistulae can lead to symptoms in 19% of patients below the age of 20 years and 63% of patients over the age of 20 years [6]. Symptoms of chest pain and dyspnea are reported in 39% and 25% of patients with CAF respectively [7].

Coexistence of multiple coronary artery-left ventricular (LV) fistulae and AHCM is very rare, and most drain from the right and left coronary arteries to the left ventricle. This phenotype is considered to represent the partial persistent embryonic myocardial sinusoids that arise from the endothelial protrusion into the inter-trabecular spaces [2]. The relationship between the two is not well established. One hypothesis suggests the apical hypertrophy could be a result of chronic volume overload of the LV through coronary artery-LV micro-fistulae; another suggests that myocardial disarray could be the cause of multiple CAF [3]. However, our patient did not have LV volume overload, as both LV dimensions on ECHO and LVEDP on cardiac catheterization were normal. Both coronary artery fistulae and AHCM can produce myocardial ischemia and angina, and their association could aggravate the ischemia. No studies have established the association between CAF and AHCM but there have been rare reports of their co-existence [3]. To our knowledge, this is the first case report on co-existing AHCM and LAD-left atrial/pulmonary artery fistula. 

Per Class I recommendations by the American Heart Association/American College of Cardiology (AHA/ACC) [8], patients with large fistulae, or those with small to moderate fistulae with complications such as ischemia, arrhythmias, or ventricular dysfunction of unexplained etiology, should undergo fistula closure. Closure may be surgical or percutaneous depending on local expertise, and is recommended especially for cases with proximal fistulae. Antiplatelet therapy is typically given for at least one year. Routine closure of small, asymptomatic CAF is not recommended (Class III) but should be followed with surveillance for symptoms and arrhythmias, along with echocardiography every three to five years to exclude the development of chamber enlargement that might alter management. (Class IIa) [8]. Studies have revealed that surgical closure of CAF has led to low mortality and morbidity and excellent long-term outcomes [9,10].

Our patient qualified for correction of the fistula since he experienced exertional symptoms, demonstrated stress-induced ischemia, and had multiple fistulae on imaging studies. Surgical closure was preferred over transcatheter closure due to the presence of multiple connections between LAD and pulmonary artery/left atrium and concomitant LAD stenosis. 

## Conclusions

CAF are mostly incidentally found in patients presenting with anginal symptoms during cardiac catheterization. Myocardial ischemia and related symptoms are due to coronary steal phenomenon and could be exacerbated by associated AHCM. Large, symptomatic CAF should be closed.
